# Contribution of Individual Risk Factor Changes to Reductions in Population Absolute Cardiovascular Risk

**DOI:** 10.1155/2014/626205

**Published:** 2014-06-05

**Authors:** Thomas Cochrane, Rachel Davey, Christopher Gidlow, Zafar Iqbal, Jagdish Kumar, Yvonne Mawby, Ruth Chambers

**Affiliations:** ^1^Centre for Research and Action in Public Health, Faculty of Health, University of Canberra, ACT 2601, Australia; ^2^Centre for Sport Health and Exercise Research, Staffordshire University, Leek Road, Stoke-on-Trent ST4 2DF, UK; ^3^Stoke-on-Trent City Council Public Health Directorate, Civic Centre, Glebe Street, Stoke-on-Trent ST4 1HH, UK

## Abstract

*Background*. Few studies have investigated individual risk factor contributions to absolute cardiovascular disease (CVD) risk. Even fewer have examined changes in individual risk factors as components of overall modifiable risk change following a CVD prevention intervention. *Design*. Longitudinal study of population CVD risk factor changes following a health screening and enhanced support programme. *Methods*. The contribution of individual risk factors to the estimated absolute CVD risk in a population of high risk patients identified from general practice records was evaluated. Further, the proportion of the modifiable risk attributable to each factor that was removed following one year of enhanced support was estimated. *Results*. Mean age of patients (533 males, 68 females) was 63.7 (6.4) years. High cholesterol (57%) was most prevalent, followed by smoking (53%) and high blood pressure (26%). Smoking (57%) made the greatest contribution to the modifiable population CVD risk, followed by raised blood pressure (26%) and raised cholesterol (17%). After one year of enhanced support, the modifiable population risk attributed to smoking (56%), high blood pressure (68%), and high cholesterol (53%) was removed. *Conclusion*. Approximately 59% of the modifiable risk attributable to the combination of high blood pressure, high cholesterol, and current smoking was removed after intervention.

## 1. Introduction

Progress in understanding the multifactorial nature of cardiovascular disease and the cumulative effect of combinations of risk factors has evolved rapidly in recent years since the pioneering research of Anderson and colleagues [1] as part of the Framingham Heart study [2]. This work is given added impetus because of the growing global burden of chronic diseases as the world's population both increases and ages [3–5]. The latter emphasises the need for greater efforts to be placed on modifying lifestyles to prevent, or at least forestall, the occurrence of some risk factors in the first place. With this objective in mind, the Department of Health in England introduced the NHS Health Checks programme in 2009 [6]. We have demonstrated previously that a local implementation of this national policy in Stoke on Trent led to around a 10% decrease in mean population cardiovascular disease (CVD) risk (32.55% to 29.57%) in an established high risk population [7]. In this paper, we investigate the contribution of change in individual risk factors to the overall change in absolute cardiovascular risk.

## 2. Methods

### 2.1. Recruitment of Practices and Patients

Full details of our study design have been given in our previous publications [7, 8]. Here we report on further detailed analysis of the individual risk factor contributions to the changes in absolute CVD risk in the study sample of 601 patients recruited from 35 general practices in Stoke on Trent, a mid-sized industrial city in the West Midlands region of England. Electronic medical records of 118,710 (57,468 female, 61,242 male) patients were screened for CVD risk, of whom 10,483 (8,521 male, 1962 female) were estimated to have a CVD risk ≥20% over the next 10 years. Diabetic patients were excluded from the study at recruitment as there was a separate diabetes-specific management programme underway in primary care across the city. Our original trial included two groups, 365 recruited to a NHS Health Check only group and 236 to a NHS Health Check plus additional lifestyle support group [7]. Since there was no significant difference in mean population CVD risk between the two groups either at baseline or at the one year follow-up point, we have merged the data and consider the contribution of individual risk factor changes to the overall population change in CVD risk.

### 2.2. Absolute Cardiovascular Risk Estimation

Absolute CVD risk was based on the Framingham 10-year CVD risk as recommended in the Joint British Societies' Guidelines [9]. In the absence of established diabetes or confirmed left ventricular hypertrophy, the estimated CVD risk for an individual patient is based on two nonmodifiable risk factors, age and gender, and three modifiable risk factors, systolic blood pressure, total to high density lipoprotein (HDL) cholesterol ratio, and smoking status. The absolute CVD risk for an individual patient is estimated as the percentage chance that that patient will experience a CVD event over the next 10 years. Using the original parametric model and associated regression coefficients, it is possible to partition out the individual risk factor contributions to the overall absolute CVD risk estimated for each patient and to examine the mechanisms for population risk reduction in more detail.

In the risk estimator used in this study, the component of risk due to the nonmodifiable combination of gender and age, *C*_GA_, is governed by an equation of the form

(1) CGA=1.2146∗gender+1.8433∗ln⁡(age) −0.3668∗gender∗ln⁡(age),

where gender is coded 1 for males and 0 for females and ln represents the natural logarithm function.

The modifiable components, *C*_SBP_, *C*_C_, and *C*_S_, due, respectively, to systolic blood pressure, total to HDL cholesterol ratio, and smoking are governed by equations of the form

(2) CSBP=1.4032∗ln⁡(systolic blood pressure),CC=0.5390 ∗ln⁡(total cholesterol to HDL cholesterol ratio),CS=0.3899∗Smoker,

where Smoker is coded 1 for a current smoker, 0 otherwise.

We have used the above formulae to calculate the component of absolute CVD risk attributable to the combined nonmodifiable risk factors and each of the three modifiable risk factors individually. For the risk factors systolic blood pressure and total to HDL cholesterol ratio, where a threshold needs to be exceeded before the factor is considered a risk, we have further divided the risk into an unmodifiable and a modifiable component. For example, a patient is only considered to have a modifiable blood pressure component of CVD risk if systolic blood pressure exceeds 140 mmHg. Thus systolic blood pressure up to 140 mmHg was taken as the unmodifiable part and anything above this threshold was taken as the modifiable component. Similarly, a patient is only considered to have a modifiable cholesterol component of CVD risk if total to HDL cholesterol ratio exceeds 4.5.

### 2.3. Changes in Modifiable Risk

For each modifiable risk factor, the change in risk was calculated as a proportion of the modifiable risk attributable to that factor so that an estimate of the proportion of modifiable risk reduced or added to could be derived for each patient. The mean population proportions were then compared with zero (i.e., no change in risk factor), using the one-sample *t*-test, to determine whether there was a significant net benefit of the intervention and, if so, to derive 95% confidence intervals for the proportion of modifiable risk reduced by the intervention. The significance level was set at *P* < 0.05.

## 3. Results

### 3.1. Characteristics of Sample Population

The baseline characteristics of the sample population are summarised in Table 1.

As can be seen from the table, this population was predominantly of older age, male, White, and of low or intermediate socioeconomic status. The risk factor profile of this high CVD risk population is shown in Table 2.

The most prevalent of the established CVD risk factors was high cholesterol (57%) followed by smoking (53%) and high blood pressure (26%). It is also worth noting that overweight or obesity was highly prevalent in this sample (76%), though this risk factor is not included explicitly in the currently (at the time of conducting this research) recommended CVD population risk estimator.

### 3.2. Components of Absolute CVD Risk

**Figure 1:**
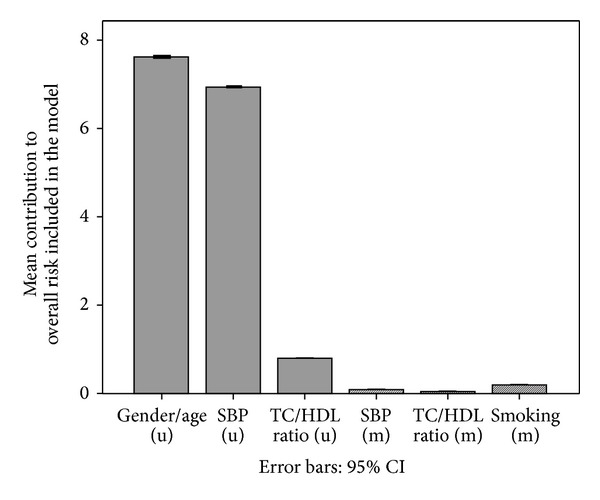
Components of absolute CVD risk. SBP: systolic blood pressure; TC/HDL: total to HDL cholesterol ratio; (u): unmodifiable; (m): modifiable.

Figure 1 summarises the various components in the absolute CVD risk estimation. The unmodifiable (u) components are shown on the left and the modifiable (m) components are shown on the right. It can be seen that the calculation is dominated by the unmodifiable risk components. Of the modifiable risk, smoking, at 57%, made the greatest contribution to total modifiable population risk followed by blood pressure, at 26%, and cholesterol, at 17%.

### 3.3. Changes in Absolute CVD Risk due to NHS Health Checks Intervention

**Figure 2:**
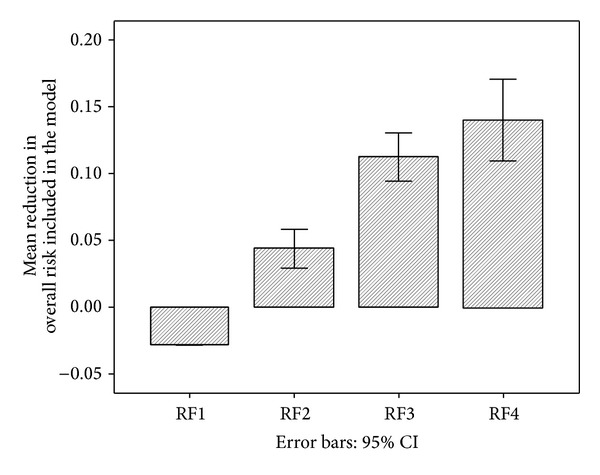
Cumulative effects of multiple risk factors. RF1: gender/age; RF2: + SBP; RF3: + TC/HDL; RF4: + smoking.

The proportion of the modifiable risk at baseline that was reduced for each of the primary risk factors is summarised in Table 3. Overall, approximately 59% of the modifiable risk at baseline was reduced after one year of intervention.  Figure 2 illustrates the cumulative benefit of treating multiple risk factors simultaneously.

The first bar shows the negative effect of age increasing by 1 year (age and gender component of risk) (RF1). This was more than compensated for by the beneficial change in the systolic blood pressure component (RF2), with additional benefit when reduction in TC/HDL ratio (RF3) and smoking components (RF4) were added.

There was wide variation in individual patient reduction in % CVD risk, ranging from −16.4 to +26.2 but no real pattern characterising those who showed no change or increased their risk and those who reduced their risk. We performed binary logistic regression analysis on the dichotomous outcome variable (0 = no change or worse, 1 = reduction in CVD risk) on predictors of age at baseline (expressed as decade = age/10 in order to obtain an odds ratio for a meaningful change in age) and categories of gender and smoking status, high blood pressure, high cholesterol, and obesity at baseline. Just two factors made a significant explanatory contribution in the model. Older patients and those who had a high blood pressure at baseline were more likely to show a reduction in CVD risk. However, the overall predictive power of the model was low, Nagelkerke's *R* ^2^ = .063.

### 3.4. Estimates of Major Vascular Disease Events Avoided in Stoke on Trent

The mean population 10-year CVD risk for females decreased from 26% at baseline to 21.2% after one year of intervention and for males mean population CVD risk decreased from 33.4% to 30.3%. Furthermore, the proportion of the male population estimated to be at high risk of experiencing a CVD related event was 13.9% (95% CI, 13.6–14.2), while that for females was 3.4% (95% CI, 3.3–3.6). Extrapolating these data to the whole of the at risk population of Stoke on Trent (~150,000, 77,500 males, 72,500 females), estimated ~450 serious CVD events could be prevented over 10 years.

## 4. Discussion

### 4.1. Key Findings

The multifactorial risk factor approach to population CVD risk reduction offers significant advantage over the single risk factor approach and has the potential to reduce the incidence of major vascular disease related events, though this would need to be confirmed in prospective longer term studies. Moreover, the success of routine screening of electronic medical records allows for a more proactive and more precisely targeted approach to the management of population CVD risk than has been available until now.

Fifty-seven percent of the modifiable CVD risk in our sample population was attributable to smoking, 26% to high blood pressure, and 17% to a high TC/HDL cholesterol ratio. The INTERHEART study [5] of potentially modifiable risk factors associated with myocardial infarction reported population attributable risks of 35.7%, 49.2%, and 17.9% for smoking, apolipoprotein B to apolipoprotein A1 ratio, and history of hypertension, respectively. Whilst the relative order of the three risk factors differs, reflecting differences in population demographics, specific measures used, and lifestyles in Stoke on Trent compared to the global population sampled in the INTERHEART study, the dominant importance of these three risk factors in the progress of cardiovascular disease is confirmed.

More importantly, perhaps, in the context of this research, approximately 59% of the modifiable CVD risk in this high risk population was removed after just one year of intervention. If replicated nationally this would represent a significant public health gain and should make a valuable contribution to reducing the burden of chronic diseases, of which the vascular disease cluster remains dominant [3, 4, 10]. This 59% reduction in modifiable CVD risk represents a substantial proportion of the 80% reported to be avoidable collectively using the nine lifestyle-related risk factors considered in the INTERHEART study. Furthermore, the relative risk, comparing estimated baseline mean population CVD risk with that after one year of follow-up, was 0.91 (29.57 versus 32.55), which is close to the value of 0.9 used by Barton et al. [11] in their modelling study to develop National Institute of Health and Clinical Excellence guidance on a public health programme aimed at preventing CVD in whole populations. Thus, our findings provide support for the value of the NHS Health Checks component of national policy for preventing CVD in the population of Stoke on Trent. This, of course, is only one part of the whole story and more needs to be done to reduce the average levels of established risk factors across the whole population, not just those already considered to be at high CVD risk [12–16]. Nonetheless, the observed changes in a deprived ex-industrial city with a poor health profile [17] such as Stoke on Trent are encouraging.

Another key finding from this research relates to the importance of unmodifiable factors (in particular gender and age) included in the risk estimator. For example, risk is unavoidably inflated for males and people of older age. This may explain why our sample population (identified on the basis of having a CVD risk of ≥20%) was predominantly male (~8 : 1 ratio of males to females) and of older mean age than would be expected from the profile of CVD events in the whole population. This may be a more important consideration than has been recognised hitherto and represents a serious limitation of the Framingham risk estimator approach to population CVD risk reduction. It is possible that our study participants included older people who, although they had a confirmed CVD risk ≥20% as per national guidelines, actually had a* modifiable* level of risk that was lower than that of younger people with an absolute risk <20%. This has implications for the overall efficiency and/or effectiveness of national policy and the optimum reduction in burden of disease achievable across the whole population. From a prevention perspective, it would be better if the risk estimator included only modifiable factors. In addition, where absolute measures are used in the calculation, it would be better if these were expressed as deviations from an established norm or only considered for values above (or below, depending on the direction of the relationship of the measure with risk) a set threshold, for example, 140 mmHg for systolic blood pressure.

These latter issues raise questions about the continuing suitability of the Framingham-based approach as the primary tool for use in the prevention of cardiovascular disease. The Framingham equations were developed from the research carried out in the town of Framingham, Massachusetts, in the United States, beginning in 1948 and still continuing today. Whilst there is no denying the importance of the factors included in the original equations, they form a limited subset of the factors now known to predispose individuals to cardiovascular disease risk and do not include factors such as obesity [18], socioeconomic deprivation [19, 20], and psychosocial stress [21, 22]. In addition, lifestyles and global socioeconomic circumstances have changed markedly since the formative period over which the original equations were developed. Furthermore, electronic communication and access to linked demographic and health care information have improved dramatically in recent years, making it feasible to develop predictive models including more locally representative populations and additional relevant variables as well as more sophisticated statistical methods, not available to the original Framingham researchers. The latter might also include multilevel approaches which allow effects of higher level factors, such as the effects of neighbourhood environments, to be included in predictive models [23–25]. Several alternative approaches have been proposed and these have been reviewed recently [26]. However, there is no strong evidence, as yet, of a significant advantage of these newer methods over the original Framingham equations [27]. Perhaps it is time for a more concerted global collaborative effort to develop a 21st century upgrade to the original Framingham equations. That said, a more important issue, as noted by Cooney et al. [26], is the underutilisation of multifactorial risk estimators and prevention guidelines in clinical practice and across the wider community.

Although not without some challenges, the roll-out of software (Oberoi Clinical Observations, Oberoi Consulting, Derby, UK) for the management of CVD risk across the majority of general practices in Stoke on Trent was achieved relatively smoothly and at reasonable cost (approximately ~*£*500 per practice per annum). This raises the possibility of a performance improvement in health care that could actually pay for itself (and more) since the societal saving in avoiding just one major cardiovascular disease event is significantly more than this. If one adds the potential to manage other chronic conditions using a similar approach, then the gains for public health could be even greater. Blumenthal [28] has advocated the setting up of health improvement communities across the United States, whereby, in return for financial, technical, and regulatory support, stakeholders in participating communities should be involved in accountable-care arrangements. In essence, the programme that we have implemented in Stoke on Trent was an embryonic trial of this basic idea. The enabling tools for the approach were improved primary care through greater efforts to screen patients, to inform them, to motivate them to take their medications (including for multiple risk factors), and to support them within their local community to make lifestyle changes that should benefit their health; funding was made available using payment for service through a local enhanced service arrangement and support for free access to additional services and a software support system that allowed each practice's electronic medical records to be searched and a cardiovascular disease risk profile to be generated, from which appropriate patients could be selected for further follow-up.

### 4.2. What Is Already Known on This Topic

Multifactorial risk factor interventions for the prevention of cardiovascular disease have been shown to be effective in well-controlled, well-funded randomised controlled trials.

Greater emphasis on prevention has been advocated to address the growing burden of chronic disorders as the world's population both grows in number and ages.

### 4.3. What This Study Adds

Routine screening of electronic medical records in general practice to estimate population CVD risk is feasible and affordable.

Coordinated high risk screening and additional support to reduce multiple risk factors simultaneously resulted in a reduction in mean population CVD risk by about 10% of baseline level. This should translate to a proportional reduction in the incidence of cardiovascular disease events.

The inclusion of nonmodifiable factors in the CVD risk estimator has the potential to bias patient selection towards older males in particular who may have lower* modifiable* CVD risk than younger patients.

### 4.4. Limitations

It was not feasible to record all constituent treatments (and compliance with these treatments) offered to the patients included in this research. Thus we were unable to attribute changes observed in blood pressure, total to HDL cholesterol ratio, and smoking habits directly to specific components of the intervention. In addition to this point, the current guideline CVD risk estimator is based predominantly on clinical measures and has limited sensitivity to other aspects of lifestyle modification that might influence CVD risk such as weight loss, increased physical activity, reduced psychosocial stress, avoidance of excessive intake of alcohol, and increased self-esteem that might result from enhanced support from within the community.

The sample population was predominantly male and of older age than would be expected from the profile of CVD events in the local population as a whole. We believe this to be due to an inadvertent sampling bias caused by the inclusion of the nonmodifiable risk factors of gender and age in the CVD risk estimator used. This makes it difficult to generalise beyond the specific population included in this evaluation of the NHS Health Checks programme as implemented in Stoke on Trent.

In assessing the components of absolute CVD risk attributable to each risk factor, we have partitioned the original parametric model into its various components and examined the contribution of each component to the overall risk included in the model. This is an intermediate stage in the actual estimation of absolute CVD risk. Thus, we were unable to attribute changes in risk factors directly to changes in absolute CVD risk estimated.

## 5. Conclusions

The NHS Health Checks programme as implemented in Stoke on Trent was successful in reducing estimated mean population cardiovascular disease risk. Around 59% of the modifiable risk attributable to the combination of high blood pressure, high cholesterol, and current smoking was removed after one year of intervention.

## Figures and Tables

**Table 1:** Baseline characteristics of sample population.

Characteristic	Category	*N* (%)
Gender	Female	68 (11.3%)
	Male	533 (88.7%)
Ethnic group	White	581 (96.7%)
	Other	20 (3.3%)
Socioeconomic status^a^	Deprived	288 (48%)
	Intermediate	195 (32%)
	More affluent	118 (20%)

Measure		Mean (SD)

Age (years)		63.7 (6.4)
Systolic blood pressure (mmHg)		145 (16.7)
Total/HDL cholesterol ratio		4.8 (1.1)
Weight (kg)		83.6 (14.1)
Body mass index (kgm^−2^)		28 (4.5)

^a^Based on the English Indices of deprivation 2010.

**Table 2:** Risk factor profile of sample population.

Risk factor	Definition	*N* (%)
High blood pressure	SBP ≥ 140 and DBP ≥ 90	158 (26%)
High cholesterol	TC/HDL ratio ≥ 4.5	344 (57%)
Current smoker	Yes	319 (53%)
Overweight	BMI ≥ 25 and BMI < 30	291 (48%)
Obese	BMI ≥ 30	166 (28%)

SBP: systolic blood pressure (mmHg); DBP: diastolic blood pressure; TC/HDL: total to high density lipoprotein cholesterol ratio; BMI: body mass index (kgm^−2^).

**Table 3:** Proportion of modifiable risk reduced for each risk factor.

	Proportion of modifiable risk reduced (%)	95% CI	*N*
Smoking	56	51–62	319***
Systolic blood pressure	68	58–77	322***
Total to HDL cholesterol ratio	53	42–64	309***

*N*: number of patients with this modifiable risk; ***P < 0.001.
